# Impact of age and immune status on the accuracy of rapid diagnostic tests for visceral leishmaniasis in Brazil

**DOI:** 10.1371/journal.pntd.0013087

**Published:** 2025-06-02

**Authors:** Mariana Lourenço Freire, Daniel Moreira de Avelar, Mariana Junqueira Pedras, Líndicy Leidicy Alves, Veronica Cardoso Santos de Faria, Lara Saraiva, Tália Santana Machado de Assis, Dorcas Lamounier Costa, Gláucia Cota

**Affiliations:** 1 Pesquisa Clínica e Políticas Públicas em Doenças Infecciosas e Parasitárias, Instituto René Rachou - Fundação Oswaldo Cruz, Belo Horizonte, Brasil; 2 Centro Federal de Educação Tecnológica de Minas Gerais, Contagem, Brasil; 3 Centro de Inteligência em Agravos Tropicais Emergentes e Negligenciados (CIATEN), Universidade Federal do Piauí, Teresina, Brazil; San Diego State University, UNITED STATES OF AMERICA

## Abstract

**Background:**

Visceral leishmaniasis (VL) represents a significant public health concern due to its high case-fatality, which poses the challenge of a timely and accurate diagnosis. Antibody-based rapid diagnostic tests (RDTs) have emerged as a disruptive innovation in recent years, by offering a diagnosis in the field, at low cost, easy to perform and with results in a few minutes. However, their performance can vary across regions and different subgroups, particularly in immunocompromised individuals. This study aimed to assess the accuracy of VL RDTs registered with the Brazilian national regulatory agency, or available through the PAHO strategic fund, considering diverse patient profiles.

**Methodology/principal findings:**

Three commercially RDTs were identified LSH Ab Eco Teste, Leishmaniasis VH Bio, and Kalazar Detect and evaluated using a well characterized panel of serum samples (n = 300) from suspected VL patients from different Brazilian regions. Sensitivity, specificity, and accuracy were determined for different patient’s ages and HIV coinfection status. Overall, RDTs exhibited lower sensitivity in children under 3 years old and HIV co-infected individuals compared to those over 3 years without HIV co-infection (p < 0.05). The agreement (Cohen’s kappa coefficient) between observers (reproducibility) and intra-test (repeatability) for all three commercial kits was excellent.

**Conclusions/significance:**

While RDTs offer desirable advantages in terms of access to diagnoses, variation on their performance imposes limits on their implementation. The performance of RDTs for VL exhibits significant differences related to age and immune status.

## Author summary

Visceral leishmaniasis (VL) is a severe disease that affects thousands of people worldwide, particularly in areas with limited healthcare resources. Early and accurate diagnosis is crucial because VL can be fatal if left untreated. Rapid diagnostic tests (RDTs) have been developed to offer fast, simple, and affordable diagnoses, making them particularly useful in remote regions. However, these tests don’t always perform equally well across different patient groups, such as children and individuals with weakened immune systems, including those co-infected with HIV. This study evaluated three commercially available RDTs for VL, focusing on their accuracy across diverse patient profiles in Brazil. The results showed that while these tests are generally reliable, they are less sensitive in children under 3 years of age and in HIV-positive patients. These findings highlight the importance of considering patient characteristics when using RDTs in the field, as certain groups may require alternative or supplementary diagnostic approaches to ensure accurate detection of the disease.

## Introduction

Visceral leishmaniasis (VL) is a severe infectious disease caused by *Leishmania* parasites transmitted by the bite of infected sand flies [1]. The zoonotic form, caused by *Leishmania infantum*, occurs in the Mediterranean basin, China, the Middle East, and South America, with dogs acting as the primary reservoir. The anthroponotic form, caused by *Leishmania donovani*, is prevalent in eastern Africa, Bangladesh, India, and Nepal [2]. According to the Global Leishmaniasis Surveillance Report, in 2022, 12.842 new VL cases (12 773 autochthonous and 69 imported) were reported to WHO. However, due to underreporting [3], it is estimated that approximately 50,000–90,000 people are annually affected across more than 80 countries, with over 90% of cases occurring in East Africa, Brazil and Indian subcontinent [4].

The clinical diagnosis of VL is challenging due to its presentation overlapping with other infections such as typhoid fever, tuberculosis, brucellosis, malaria, mononucleosis syndrome, AIDS and certain malignant hematological diseases [5,6]. Furthermore, since VL is lethal, rapid, and accurate laboratory diagnosis is imperative. However, in regions where the disease is endemic, the available diagnostic tests may be limited and depend more on the resources and infrastructure available rather than the accuracy of the employed method. Currently, diagnostic options in Brazil encompass direct parasitological examination, polymerase chain reaction (PCR), and serological assays, mainly immunochromatographic rapid diagnostic tests (RDTs) [7]. RDTs, especially those using recombinant antigen K39 (rK39), represented a breakthrough in VL diagnosis context [8]. The advantages of RDTs include low cost, quick results, ease of use, and simplicity, making them the most widely used, cost-effective tool and the first choice for point-of-care diagnosis of the VL in endemic areas [9,10]. Their performance has been considered high, but it may vary depending on several factors, including epidemiological dynamics (such as circulating *Leishmania* species), intrinsic test characteristics (including the antigen utilized and the testing platform), and the clinical condition of the patient [10–13]. Performance for different RDTs, ranging from 67.6% to 100% for sensitivity and 70% to 100% for specificity, has been reported [10,11,14,15]. Lower sensitivity is frequently reported in some endemic regions, such as Brazil and East Africa, compared to the Indian subcontinent [10]. Results obtained with different diagnostic kits in the past have already indicated that immunosuppressed individuals, particularly those infected by HIV, are ones for whom RDTs admittedly fail to meet the required standards [12,13]. In relation to children, although hypotheses of lower performance have been raised, these data have not yet been widely evaluated in the Americas [16].

The clinical diagnosis of VL is challenging due to its presentation overlapping with other infections such as typhoid fever, tuberculosis, brucellosis, malaria, mononucleosis syndrome, AIDS and certain malignant hematological diseases [5,6]. Furthermore, since VL is lethal, rapid, and accurate laboratory diagnosis is imperative. However, in regions where the disease is endemic, the available diagnostic tests may be limited and depend more on the resources and infrastructure available rather than the accuracy of the employed method. Currently, diagnostic options in Brazil encompass direct parasitological examination, polymerase chain reaction (PCR), and serological assays, mainly immunochromatographic rapid diagnostic tests (RDTs) [7]. RDTs, especially those using recombinant antigen K39 (rK39), represented a breakthrough in VL diagnosis context [8]. The advantages of RDTs include low cost, quick results, ease of use, and simplicity, making them the most widely used, cost-effective tool and the first choice for point-of-care diagnosis of the VL in endemic areas [9,10]. Their performance has been considered high, but it may vary depending on several factors, including epidemiological dynamics (such as circulating *Leishmania* species), intrinsic test characteristics (including the antigen utilized and the testing platform), and the clinical condition of the patient [10–13]. Performance for different RDTs, ranging from 67.6% to 100% for sensitivity and 70% to 100% for specificity, has been reported [10,11,14,15]. Lower sensitivity is frequently reported in some endemic regions, such as Brazil and East Africa, compared to the Indian subcontinent [10]. Results obtained with different diagnostic kits in the past have already indicated that immunosuppressed individuals, particularly those infected by HIV, are ones for whom RDTs admittedly fail to meet the required standards [12,13]. In relation to children, although hypotheses of lower performance have been raised, these data have not yet been widely evaluated in the Americas [16].

Given the critical need to guide public health interventions and optimize resource allocation in endemic countries, a comprehensive understanding of test performance is paramount and should be conducted in all endemic regions. Unfortunately, there is a scarcity of published scientific data and no requirement from health agencies regarding minimum performance for RDTs in specific subgroups. For other American countries, unlike Brazil, only the immunochromatographic test Kalazar Detect has secured availability, procured through the strategic fund of the Pan American Health Organization (PAHO). It is a kit licensed exclusively for serum sample use, posing logistical challenges. To gain insight into the current landscape of rapid serological diagnostic options for visceral leishmaniasis in the Americas and assess their performance in special populations, this study aimed to evaluate the accuracy of several RDTs available in Brazil. A representative panel of samples from diverse geographical regions of the country was used, including those from children, adults, and immunosuppressed patients.

## Materials and methods

### Ethics statement

This study was conducted in accordance with the Research Ethics Committee and was approved by the Instituto René Rachou (IRR)/ Fundação Oswaldo Cruz (CAAE 65354722.6.0000.5091– Approval number 6.630.798). Written informed consent was obtained from all participants, or their parents/guardians, before sample collection during the original clinical studies. Dates were accessed from January 10–30, 2024 and were fully anonymized before analysis. Confidentiality was maintained by assigning a unique study code to each sample, ensuring no confidential information was disclosed. This diagnostic accuracy study utilized a stored panel of serum samples from patients suspected of VL. The study was conducted in accordance with the Standards for Reporting of Diagnostic Accuracy Studies (STARD) guidelines [17] available in S1 Appendix.

### Sample included

Serum samples were sourced from five biorepositories gathered in three distinct Brazilian federated units: Minas Gerais, Piauí, and Bahia. Serum samples from patients with a clinical suspicion of VL were included if clinical and demographic data, along with parasitological and molecular test results, were available. All samples were kept frozen at -70°C. Samples were excluded if the available volume was less than 300µL.

### Rapid serological diagnostic tests

A search for tests registered in Brazil for VL diagnosis was performed on February 7, 2024, via the electronic database of the Brazilian Health Surveillance Agency (ANVISA), using the following keywords: ‘*Leishmaniasis*, *Leishmania*, *Leish*, and *rK-39*’. After identifying tests with active registration, verification of commercial availability was carried out by direct contact with the manufacturers via web pages, e-mail, or telephone. Of the six tests found registered in Brazil, only the LSH Ab Eco Teste (Eco Diagnóstica Ltda) and Leishmaniasis VH Bio (Quibasa Química Básica Ltda) tests were commercially available. In addition, the Kalazar Detect test (INBIOS International Inc. – United States), was included, as it is listed among the products of the Pan American Health Organization (PAHO) strategic fund, which makes it accessible for purchase and use by endemic countries in the Americas.

Thus, in this study, the Ab Eco Test (Eco Diagnóstica Ltda), the Leishmaniasis VH Bio (Quibasa Química Básica Ltda), and the Kalazar Detect Test (INBIOS International Inc.) were included, all of which are rapid tests based on the qualitative detection of antibodies against the recombinant antigen (rK39), specific to parasites of the *L. donovani complex.* Some characteristics of the included tests are presented in Table 1.

**Table 1:** Main properties of evaluated commercial tests.

Diagnostic test	Batch	Validity	Serum sample	Time to interpret results
LSH Ab Eco Teste (Eco Diagnóstica)	202403002	11/02/2025	20µL	10 min
Leishmaniose VH Bio (Quibasa)	13.2	01/2024	10µL	10 to 20 min
Kalazar Detect (INBIOS International Inc.)	CG6170	07/31/2025	20µL	10 min

### Sample size, characteristics of sera and study design

To estimate the minimum sample size of the serum panel, a sensitivity of at least 95%, with a confidence interval of 95% for an RDT for VL screening (WHO, 2011), an alpha error of 5%, and a power of 80% were considered. The minimum total number (cases and controls) of sera for comparing proportions in the same sample was calculated to be 150.

The VL case definition adopted was based on the presence of clinical manifestations suggestive of the disease, associated with the identification of *Leishmania* spp. in bone marrow aspirates by direct examination, in vitro culture isolation of the parasite, or the detection of *Leishmania* spp. by PCR in blood or bone marrow samples, which were assumed as the reference standard for VL diagnosis. All sera used as non-cases were obtained from the original studies involving subjects presenting clinical manifestations compatible with VL. These individuals had VL ruled out and received confirmed diagnoses of other conditions, including leukemia, typhoid fever, bone marrow aplasia, infective endocarditis, sickle cell anemia, schistosomiasis, malaria, Chagas disease, or liver disease.

To assess the performance of the tests in specific subgroups, a minimum of 75 case samples from each group of interest was gathered, along with a control group consisting of 75 samples from adults and children with a confirmed diagnosis of a disease other than VL. Therefore, the serum panel comprised 300 samples, including 225 positives for VL (cases) and 75 negatives for VL (non-cases). The VL patient subgroups were divided as follows: a) 75 samples from children up to 3 years old, b) 75 samples from individuals older than 3 years and 1 month, and c) 75 samples from individuals with HIV/VL coinfection. The age cutoff of three years was selected based on the findings of Freire et al. (2019) [16], who analyzed different age thresholds and demonstrated a statistically significant difference in the performance of the Kalazar Detect test between children younger than three years and older individuals.

Tests were done according to the manufacturer’s instructions, using the sample, buffer volume and the reading time indicated in each manufacturer’s instructions. Two researchers, blind to the sample condition (case or non-case), performed the tests sequentially and conducted independent visual readings. Disagreements were resolved by a third researcher. Tests from the same manufacturer were carried out on the same day.

### Statistical analysis

Sensitivity, specificity, and accuracy were calculated using two-by-two contingency tables with exact binomial 95% confidence intervals (95% CI). Sensitivity was calculated as the number of patients with VL who tested positive divided by the total number of patients with VL, while specificity was calculated as the number of non-VL patients who tested negative divided by the total number of non-VL patients. Accuracy was defined as the proportion of patients presenting a correct test result and was calculated as the number of patients with VL who tested positive plus the number of non-VL patients who tested negative divided by the total number of patients tested. For the comparison of two proportions (from independent samples), expressed as percentages, the Chi-squared test was used. These analyses were performed by using MedCalc version 15.0 for Windows (Ostend, Belgium).

Cohen’s kappa coefficient was calculated by GraphPad Quick Calcs (San Diego, CA) to assess the agreement between the results of each test, with values interpreted according to Landis and Koch: 1.00–0.81: excellent, 0.80–0.61: good, 0.60–0.41: moderate, 0.40–0.21: weak, and 0.20–0.00: negligible agreement. Furthermore, the agreement rate between tests was calculated by considering the sum of true positives and true negatives in two tests, divided by the total number of evaluated patients.

The repeatability of each test was assessed by determining the degree of agreement between the results of three successive measurements of the same sample, conducted under the same conditions on the same day.

## Results

### Participants’ characteristics

Sixty-eight percent of the 300 suspected VL patients were male, with a median age of 23.3 years (ranging from one month to 80 years). Among children up to 3 years old, 60.3% were male, with a median age of one year and six months (ranging from one month to two years and nine months). Among individuals older than 3 years and 1 month, 64% were male, with a median age of 23 years (ranging from 3 years and 1 month to 68 years).

For HIV-coinfected individuals, the median age was 40 years (ranging from 21 to 80 years), and 80.9% of this group were male. Although not all HIV-coinfected individuals had their CD4 count reported, among the 65 patients with available information, 70.8% had a CD4 count below 200 cells/mm³. The characteristics of the patients included in the validation study are detailed in S2 Appendix.

### Performance of RDTs

The sensitivity, specificity, and accuracy of each RDT are presented in Table 2 for the different subgroups. Among children, the Kalazar Detect test demonstrated a sensitivity of 82.7% (95% CI: 72.6–89.6), which was significantly higher than the sensitivity of the *Leishmaniose* VH Bio test at 62.7% (95% CI: 51.4–72.7) for children under 3 years of age (p = 0.006). In this subgroup, all tests exhibited significantly lower sensitivity (p < 0.05) compared to those older than this age. Notably, all tests reached a sensitivity of 93.3% (95% CI: 85.3–97.1) in patients over 3 years and 1 month old. In individuals with HIV infection, the sensitivity of all tests was significantly lower compared to both children under 3 years old (p < 0.05) and patients older than 3 years (p < 0.0001). Regarding specificity, the LSH Ab Eco Teste and Leishmaniose VH Bio showed the highest values at 94.7% (95% CI: 87.1–97.9), followed closely by Kalazar Detect at 93.3% (95% CI: 85.3–97.1), with no significant differences among the tests. In terms of overall accuracy, Kalazar Detect achieved the highest accuracy at 90.2% (95% CI: 85.6–93.4), followed by LSH Ab Eco Teste at 86.7% (95% CI: 81.6–90.5) and Leishmaniose VH Bio at 83.6% (95% CI: 78.2–87.8).

**Table 2:** Performance of the human visceral leishmaniasis RDTs stratified according to the subgroups.

Performance (CI 95%)	LSH Ab Eco Teste (Eco Diagnóstica)	Leishmaniose VH Bio (Quibasa)	Kalazar Detect (INBIOS International)
Sensitivity in children ≤ 3 years old (n = 75)	72.0 (60.9–80.9) (54/75)	62,7 (51.4–72.7) (47/75)	82.7 (72.6–89.6) (62/75)
Sensitivity in patients > 3 years and 1 month old (n = 75)	93.3 (85.3–97.1) (70/75)	93.3 (85.3–97.1) (70/75)	93.3 (85.3–97.1) (70/75)
Sensitivity in individual with LV/HIV coinfection² (n = 75)	53.3 (42.2–64.2) (40/75)	46.7 (35.8–57.8) (35/75)	57.3 (46.1–67.9) (43/75)
Specificity (n = 75)	94.7 (87.1–97.9) (71/75)	94,7 (87.1–97.9) (71/75)	93.3 (85,3–97.1) (70/75)
Accuracy (n = 225)	86.7 (81.6–90.5) (195/225)	83.6 (78.2–87.8) (188/225)	90.2 (85.6–93.4) (202/225)

The sensitivity of the evaluated tests varied according to age, with notably lower sensitivity among children under two years compared to those aged between 2 and 3 years. (Table 3).

**Table 3:** Visceral leishmaniasis RDTs’ sensitivity stratified according to age.

Sensitivity (CI95%)	LSH Ab Eco Teste (Eco Diagnóstica)	Leishmaniose VH Bio (Quibasa)	Kalazar Detect (INBIOS International)
Children ≤ 3 years old (n = 75)	72.0 (60.9–80.9) (54/75)	62,7 (51.4–72.7) (47/75)	82.7 (72.6–89.6) (62/75)
a.Children < 2 years old(n = 49)	67.4 (53.4–78.8) (33/49)	57,1 (43.3–69.9) (28/49)	77.6 (64.1–86.9) (38/49)
b.Children 2–3 years old (n = 26)	80.8 (62.1–91.5) (21/26)	73,1 (53.9–86.3) (19/26)	92.3 (75.9–97.9) (24/26)

The agreement between the two observers was classified according to kappa index (k) as almost perfect for both Kalazar Detect (INBIOS International) and Leishmaniose VH Bio tests (k = 1.000). A similar level of agreement was observed for LSH Ab Eco Teste, a kappa index of 0.980 (95% CI: 0.957–1.000). In this study there were no indeterminate or missing results.

Repeatability was evaluated using five random samples (four positive and one negative), with the tests performed sequentially three times. For all tests, the results were 100% concordant.

The agreement between the tests, assessed in pairs, is detailed in S3 Appendix. Generally, the observed agreement ranged from substantial to almost perfect. The notable exception was the comparison between the VH Bio test (Quibasa) and Kalazar Detect (INBIOS International) for the subgroup of children under 3 years old, where only moderate agreement was observed.

## Discussion

This study presents the current panorama of the performance of VL tests available in Brazil, conducted under standardized conditions using a panel of serum from individuals suspected of having VL and different ages and HIV co-infection status. The main contribution of this study is the confirmation of the impact of age and the presence of HIV co-infections on the performance of VL Rapid Diagnostic Tests (RDTs). The significantly lower test sensitivity in HIV-infected individuals and children under 3 years old was confirmed for different commercial kits. Limited research has evaluated the performance of rapid tests in individuals with VL-HIV co-infection in Brazil [13,18,19], and even fewer studies have focused on their use in children. This is particularly significant given that approximately 9% of VL cases reported in Brazil are confirmed with HIV co-infection, and over 30% affect children under 4 years old [20,21].

Notably, all rapid tests currently registered and commercially available in Brazil have not undergone prior validation studies. Among the kits evaluated in this study, only Kalazar Detect had performance described in the Brazilian population with variable sensitivity (72.4-90%) and specificity (82–100%) [16,19,22–25].

The three rapid tests included in this study exhibit high similarity in their fundamental properties, utilizing the same antigen (rk39), the same protein conjugated to colloidal gold and results available within 10 minutes. Overall, they also showed comparable performance. A significant difference was observed only for children with less than 3 years among Kalazar Detect and Leishmaniose VH Bio. For all three tests, sensitivity among individuals over 3 years of age and specificity reached the acceptable ranges proposed by the World Health Organization for the diagnosis of VL: 95% sensitivity and 96% specificity (WHO - Target Product Profiles, 2024). A high similarity between the reagent strips for Kalazar Detect and LSH Ab Eco Teste was observed, but a significant difference in the instructional leaflets provided by the respective manufacturers and which come with the kits: while LSH Ab Eco Teste is presented for use in both serum and digital pulp whole blood, Kalazar Detect is informed as exclusively for serum, a difference with important operational implications. Serological tests with rapid visual reading that can be performed using digital pulp blood without requiring centrifugation provide an extraordinary advantage in terms of execution in remote areas, without laboratory infrastructure and by professionals with minimal qualification, representing a major advance for diagnosis and, consequently, for disease control. It is important to note that at least one study compared the performance of Kalazar Detect in digital blood and serum and did not identify any differences in the accuracy [19].

A statistically significant difference in the performance of RDT was observed comparing individuals with and without HIV infection, as previously demonstrated by others [13,26,27]. This lower accuracy can be attributed to the impaired ability of HIV-infected patients to produce antibody titters detectable by these tests [28]. In this subgroup, possibly, the strategy with the greatest potential to replace time-consuming and invasive parasitological tests would be platforms based on antigen detection or molecular tests, such as PCR [13].

The low RDT’s performance among children, as the result of an immature immune system [29], is worrying given the large proportion of this age subgroup among cases reported in the Americas. It is worth mentioning that almost all of the children group’ samples came from the northeast region of Brazil, which makes it necessary to extend this evaluation to serum from children with VL suspicion living in other regions. There are few studies addressing this phenomenon in the different endemic regions. Furthermore, the age groups in comparison vary between studies, according to the epidemiological pattern of each area, which limits the extrapolation of findings. In Iran, where more than 90% of VL cases occur in children [30], a validation study of three rapid tests demonstrated sensitivities ranging from 70% to 81% [31]. In Brazil, Freire et al. (2019) observed lower accuracy in children aged 3 years only for Kalazar Detect [16]. In a study carried out by CRUZ et al. (2006) in the Mediterranean region, the performance of the rapid test did not differ between individuals over and below 10 years old [32]. In this study, a particularly low RDT‘s sensitivity was observed among children between two and three years of age, however, the small sample representation of children under 1 year of age may have compromised the reliable observation of test performance in this age subgroup.

The main strengths of this study are the use of a well-characterized and size-dimensioned panel of samples, ensuring the same conditions for the three kits evaluated, namely blinding, independent execution, and the same reference criteria for cases and non-cases. However, a limitation of our study was the use of frozen samples, which poses a risk of loss of viability due to antibody degradation over storage time. Another potential limitation of this study, what applies equally to all tests and is inherent to the VL condition, refers to the lack of a perfect reference diagnostic test for VL (gold standard). To mitigate this, a composite VL definition was adopted (including not only parasitological tests results but also molecular tests) and for all the non VL cases included, in addition to negative VL tests, confirmation of one alternative condition was also required. Another point is the inability to stratify the control group by age or HIV status due to sample size constraints. Despite this limitation, this group is highly representative of patients who are typically tested for suspected VL, including both HIV-positive and HIV-negative individuals. According to the WHO, achieving the Sustainable Development Goals by 2030 primarily requires the development of more sensitive rapid diagnostic tests for VL [33]. However, while RDTs appear to be accurate for diagnosing adults with non-immunocompromised patients, a significant challenge is their lower performance in HIV co-infected patients and small children (up to 3 years old). It is crucial to disseminate that a negative RDT result cannot rule out VL in individuals living with HIV and children under 3 years old.

## Supporting information

S1 Appendix STARD checklist and flow-diagram for reporting of studies of diagnostic accuracy. (DOCX)

S2 Appendix Detailed Information on Patient Characteristics for Validation Study. (PDF)

S3 Appendix Agreement between the RDTs evaluated according to the subgroups. (DOCX)
