# Supplementary material for: Impact of age and immune status on the accuracy of rapid diagnostic tests for visceral leishmaniasis in Brazil
Source: PLoS Negl Trop Dis. 2025 Jun 2;19(6):e0013087. doi: 10.1371/journal.pntd.0013087 (PMC12129180; doi:10.1371/journal.pntd.0013087)
Supplement: S2 Appendix — (PDF) [file pntd.0013087.s002.pdf]

S2 file. Detailed Information on Patient Characteristics for Validation Study

N°	Origin	Group	M/F	Age	VL	HIV	CD4 (cell/mm³)	Diagnosis	Bioclin	Kalazar Detect	ECO
1	MG	LV/HIV	M	41	1	1	108	VL	1	1	1
2	MG	VL > 3 years and 1 month	M	36	1	0		VL	1	1	1
3	MG	non-cases	F	44.12	0	0		Schistosomiasis	0	0	0
4	Piauí	non-cases	F	5.4	0	0		Sickle Cell Anemia	0	0	0
5	MG	LV/HIV	F	33	1	1	60	VL	1	1	1
6	Piauí	VL < 3 years	M	1.8	1	0		VL	0	0	0
7	Piauí	LV/HIV		51.1	1	1	164	VL	1	1	1
8	BH	VL < 3 years	M	1.8	1	0		VL	0	1	0
9	Piauí	VL < 3 years	F	0.5	1	0		VL	0	1	1
10	MG	LV/HIV	M	44	1	1	483	VL	0	1	0
11	Piauí	LV/HIV		44.8	1	1		VL	1	1	1
12	Piauí	LV/HIV		44.7	1	1	162	VL	0	0	0
13	MG	VL > 3 years and 1 month	M	7.2	1	0		VL	1	1	1
14	BH	non-cases	F	>18 ano	0	0		typhoid	0	0	0
15	Piauí	VL > 3 years and 1 month	F	6.3	1	0		VL	1	1	1
16	MG	LV/HIV	M	37	1	1	142	VL	1	1	1
17	MG	non-cases	M	>18 ano	0	0		Leukemia	0	0	0
18	MG	VL > 3 years and 1 month	M	39	1	0		VL	1	1	1
19	BH	VL > 3 years and 1 month	F	6.7	1	0		VL	1	1	1
20	MG	non-cases	F	>18 ano	0	0		Lymphoma	0	0	0
21	Piauí	LV/HIV		37.2	1	1	58	VL	1	1	1
22	Piauí	VL < 3 years	M	0.9	1	0		VL	1	1	1
23	MG	LV/HIV	F	34	1	1	6	VL	1	1	1
24	BH	non-cases	M	11.6	0	0		Schistosomiasis	0	0	0
25	Piauí	LV/HIV		25	1	1	629	VL	0	0	0
26	Piauí	VL < 3 years	F	1.4	1	0		VL	1	1	1
27	MG	LV/HIV	M	51	1	1	10	VL	1	1	1
28	Piauí	VL > 3 years and 1 month	M	7.12	1	0		VL	1	1	1
29	Piauí	VL > 3 years and 1 month	M	9.6	1	0		VL	1	1	1
30	MG	LV/HIV	F	37	1	1		VL	0	1	0
31	Piauí	VL > 3 years and 1 month	F	60.6	1	0		VL	1	1	1
32	MG	VL > 3 years and 1 month	M	23	1	0		VL	1	1	1
33	Piauí	LV/HIV		45	1	1	182	VL	0	1	1
34	Piauí	VL < 3 years	M	2.5	1	0		VL	1	1	1
35	MG	non-cases	F	42	0	1		hiv	0	0	0
36	Piauí	VL > 3 years and 1 month	F	19	1	0		VL	1	1	1
37	Piauí	VL > 3 years and 1 month	M	16.1	1	0		VL	1	1	1
38	Piauí	VL < 3 years		0.6	1	0		VL	1	1	1
39	BH	VL < 3 years	M	1.10	1	0		VL	0	1	0
40	Piauí	VL < 3 years	M	1.9	1	0		VL	1	1	1
41	Piauí	VL > 3 years and 1 month	M	46.1	1	0		VL	1	1	1
42	Piauí	VL < 3 years		0.4	1	0		VL	1	1	1
43	Piauí	VL < 3 years	M	2.6	1	0		VL	1	1	1
44	Piauí	LV/HIV		26	1	1	3	VL	0	0	0
45	BH	non-cases	M	8.3	0	0		Bone marrow aplasia	0	0	0
46	MG	VL > 3 years and 1 month	M	33	1	0		VL	1	1	1
47	MG	VL > 3 years and 1 month	F	35	1	0		VL	1	1	1
48	Piauí	VL < 3 years	F	1.3	1	0		VL	0	1	1
49	Piauí	VL < 3 years		0.7	1	0		VL	1	1	1
50	Piauí	non-cases	M	1.6	0	0		Urinary Tract Infection + Bacterial Pneumonia	0	0	0
51	MG	LV/HIV	M	45	1	1	14	VL	1	1	1
52	Piauí	VL < 3 years		1.8	1	0		VL	1	1	1
53	Piauí	VL > 3 years and 1 month	M	16.1	1	0		VL	1	1	1

54	Piauí	VL < 3 years	M	1.9	1	0		VL	1	1	1
55	MG	non-cases	F	34	0	1		Endocrine metabolic or deficiency disease	0	0	0
56	Piauí	LV/HIV		45.9	1	1	12	VL	1	1	1
57	MG	LV/HIV	M	53	1	1	45	VL	0	0	0
58	Piauí	VL < 3 years	M	0.1	1	0		VL	1	0	1
59	MG	non-cases	M	>18 ano	0	1		Fungal disease	0	0	0
60	MG	LV/HIV	M	53	1	1	44	VL	0	0	0
61	Piauí	LV/HIV		54.1	1	1	543	VL	1	1	1
62	BH	VL < 3 years	M	1.4	1	0		VL	0	1	1
63	Piauí	VL < 3 years	M	2.8	1	0		VL	1	1	1
64	BH	VL < 3 years	M	2.5	1	0		VL	1	1	1
65	BH	VL < 3 years	F	2.6	1	0		VL	1	1	1
66	MG	LV/HIV	M	57	1	1	58	VL	0	0	0
67	MG	non-cases	M	33	0	0		Adult Still's Disease	0	0	0
68	Piauí	LV/HIV		80.7	1	1	39	VL	0	0	0
69	MG	non-cases	F	40	0	1		Secondary to HIV	0	0	0
70	BH	VL < 3 years	M	1.9	1	0		VL	1	1	1
71	Piauí	VL < 3 years		0.4	1	0		VL	1	1	1
72	BH	VL < 3 years	F	2.9	1	0		VL	1	1	1
73	Piauí	LV/HIV		31.5	1	1	355	VL	0	0	0
74	BH	non-cases	M	4.3	0	0		lymphoma	0	0	0
75	MG	LV/HIV	M	36	1	1	90	VL	0	0	0
76	Piauí	VL > 3 years and 1 month	M	3.12	1	0		VL	0	0	0
77	MG	LV/HIV	F	28	1	1	17	VL	0	0	0
78	Piauí	VL < 3 years		2.3	1	0		VL	1	1	1
79	MG	LV/HIV	M	38	1	1	89	VL	1	1	1
80	Piauí	VL > 3 years and 1 month	F	5.4	1	0		VL	1	1	1
81	Piauí	VL > 3 years and 1 month	M	43.12	1	0		VL	1	1	1
82	BH	VL < 3 years	M	2.1	1	0		VL	1	1	1
83	Piauí	LV/HIV		64.5	1	1	13	VL	1	1	1
84	Piauí	VL > 3 years and 1 month	F	8.3	1	0		VL	1	1	1
85	MG	VL > 3 years and 1 month	M	45.6	1	0		VL	1	1	1
86	Piauí	VL < 3 years	M	0.9	1	0		VL	1	1	1
87	Piauí	VL < 3 years		1.1	1	0		VL	0	0	0
88	MG	non-cases	M	>18 ano	0	0		Leukemia	0	0	0
89	Piauí	VL > 3 years and 1 month	F	20.8	1	0		VL	1	1	1
90	MG	non-cases	M	25.3	0	0		Lupus	0	0	0
91	Piauí	VL < 3 years	F	0.8	1	0		VL	1	1	1
92	Piauí	LV/HIV		39.4	1	1	434	VL	1	1	1
93	Piauí	VL < 3 years		2.1	1	0		VL	1	1	1
94	Piauí	non-cases	M	16.4	0	0		Acute Chagas disease	0	0	0
95	Piauí	VL > 3 years and 1 month	F	20.12	1	0		VL	1	1	1
96	Piauí	LV/HIV		39.3	1	1	21	VL	0	0	0
97	Piauí	VL < 3 years	M	1.1	1	0		VL	1	1	1
98	Piauí	VL < 3 years	M	1.6	1	0		VL	1	1	1
99	Piauí	LV/HIV		46.6	1	1	710	VL	1	1	1
100	MG	non-cases	F	74.2	0	0		Temporal arthritis	0	0	0
101	MG	LV/HIV	M	45	1	1	191	VL	0	0	0
102	MG	non-cases	M	40	0	0		cirrhosis + viral infection	0	0	0
103	Piauí	LV/HIV		35.8	1	1	3	VL	0	0	0
104	BH	VL < 3 years	F	2.2	1	0		VL	1	1	1
105	MG	non-cases	M	44	0	0		Schistosomiasis	0	0	0
106	Piauí	VL < 3 years	F	0.9	1	0		VL	1	1	1
107	Piauí	VL < 3 years		1.2	1	0		VL	0	0	0
108	BH	VL > 3 years and 1 month	F	3.6	1	0		VL	1	1	1

109	MG	VL > 3 years and 1 month	M	5.11	1	0		VL	1	1	1
110	Piauí	non-cases	M	2.12	0	0		Kidney tumor (Neuroblastoma)	0	0	0
111	Piauí	VL < 3 years		1.2	1	0		VL	1	1	1
112	Piauí	VL > 3 years and 1 month	M	21.7	1	0		VL	1	1	1
113	MG	VL < 3 years	M	1.10	1	0		VL	1	1	1
114	BH	non-cases	F	6.1	0	0		Bone marrow aplasia	0	0	0
115	MG	non-cases	M	40	0	1		Fungal disease	0	0	0
116	BH	non-cases	F	14.8	0	0		Lupus	0	0	0
117	MG	non-cases	M	42	0	1		Chronic fibrosing liver disease	0	0	0
118	Piauí	LV/HIV		29.7	1	1	4	VL	0	0	0
119	BH	VL < 3 years	F	2.4	1	0		VL	1	1	1
120	BH	VL > 3 years and 1 month	F	14.8	1	0		VL	1	1	1
121	BH	non-cases	M	9.12	0	0		Leukemia	0	0	0
122	BH	VL < 3 years	F	1.6	1	0		VL	0	0	0
123	MG	LV/HIV	M	40	1	1	242	VL	0	0	0
124	Piauí	VL < 3 years	M	0.9	1	0		VL	1	1	1
125	Piauí	LV/HIV		41.8	1	1		VL	0	1	1
126	Piauí	VL < 3 years	F	0.2	1	0		VL	1	1	1
127	Piauí	VL > 3 years and 1 month	M	27.1	1	0		VL	1	1	1
128	Piauí	VL < 3 years		2.8	1	0		VL	0	1	1
129	MG	LV/HIV	M	30	1	1	122	VL	0	0	0
130	Piauí	non-cases	M	36.11	0	0		Liver failure	1	1	1
131	Piauí	LV/HIV		34.9	1	1	635	VL	1	1	1
132	MG	non-cases	M	41	0	0		arbovirus	0	0	0
133	MG	non-cases	M	>18 ano	0	0		Bone marrow aplasia	0	0	0
134	Piauí	VL > 3 years and 1 month	M	21.3	1	0		VL	1	1	1
135	MG	VL > 3 years and 1 month	F	24	1	0		VL	1	1	1
136	Piauí	VL < 3 years		0.6	1	0		VL	1	1	1
137	BH	non-cases	M	4.7	0	0		Bone marrow aplasia	0	0	0
138	Piauí	VL > 3 years and 1 month	M	30.12	1	0		VL	1	0	1
139	Piauí	LV/HIV		48.5	1	1	80	VL	1	1	1
140	MG	LV/HIV	M	29	1	1	37	VL	0	0	0
141	MG	VL > 3 years and 1 month	M	16.1	1	0		VL	1	1	1
142	Piauí	non-cases	M	66.11	0	0		Fever of unclear etiology	0	0	0
143	MG	non-cases	M	28.7	0	0		Mycobacteriosis	0	0	0
144	MG	non-cases	M	39	0	1		Fungal disease	0	0	0
145	MG	VL > 3 years and 1 month	M	20	1	0		VL	1	1	1
146	MG	LV/HIV	M	27	1	1		VL	0	0	0
147	BH	non-cases	F	10.7	0	0		Typhoid fever	0	0	0
148	MG	LV/HIV	M	53	1	1	92	VL	1	1	1
149	BH	non-cases	F	4.6	0	0		Leukemia	0	0	0
150	BH	non-cases	F	7.4	0	0		Bone marrow aplasia	0	0	0
151	Piauí	VL < 3 years	F	1.5	1	0		VL	0	1	0
152	Piauí	VL > 3 years and 1 month	M	36.10	1	0		VL	1	1	1
153	Piauí	VL > 3 years and 1 month	M	3.8	1	0		VL	1	1	1
154	MG	VL > 3 years and 1 month	F	23.3	1	0		VL	1	1	1
155	MG	LV/HIV	M	35	1	1	145	VL	1	1	1
156	MG	VL > 3 years and 1 month	F	36	1	0		VL	1	1	1
157	MG	VL > 3 years and 1 month	M	18	1	0		VL	1	1	1
158	MG	LV/HIV	F	44	1	1		VL	1	1	1
159	MG	LV/HIV	M	25	1	1	356	VL	0	1	0
160	Piauí	LV/HIV		43.5	1	1		VL	1	1	1
161	Piauí	VL > 3 years and 1 month	F	37.5	1	0		VL	1	1	1

162	Piauí	VL < 3 years		1.6	1	0		VL	0	1	0
163	MG	LV/HIV	M	38	1	1	46	VL	1	1	1
164	Piauí	VL > 3 years and 1 month	F	3.11	1	0		VL	1	1	1
165	Piauí	VL < 3 years	M	2.6	1	0		VL	1	1	1
166	MG	non-cases	M	45	0	1		Other Infectious Diseases	0	0	0
167	MG	LV/HIV	F	47	1	1	132	VL	0	0	0
168	MG	VL < 3 years	M	1.9	1	0		VL	0	0	0
169	MG	LV/HIV	M	61	1	1	206	VL	0	0	0
170	Piauí	VL < 3 years		2.4	1	0		VL	1	1	1
171	MG	VL > 3 years and 1 month	F	14	1	0		VL	1	1	1
172	MG	non-cases	M	31	0	1		Other Infectious Diseases	0	0	0
173	Piauí	VL > 3 years and 1 month	M	31.9	1	0		VL	1	1	1
174	MG	non-cases	M	31.12	0	0		Lymphoma	1	1	1
175	Piauí	VL > 3 years and 1 month	F	20.9	1	0		VL	1	1	1
176	Piauí	non-cases	M	32.11	0	0		Liver disease	0	0	0
177	Piauí	LV/HIV		32.9	1	1	19	VL	1	1	1
178	BH	VL < 3 years	F	2.5	1	0		VL	1	1	1
179	BH	VL < 3 years	M	1.7	1	0		VL	0	0	0
180	Piauí	VL > 3 years and 1 month	F	14.12	1	0		VL	1	1	1
181	Piauí	LV/HIV		25.6	1	1		VL	1	1	1
182	MG	non-cases	M	32.5	0	0		Hodgkin's disease	0	0	0
183	Piauí	VL > 3 years and 1 month	F	30.2	1	0		VL	1	1	1
184	MG	LV/HIV	F	36	1	1	265	VL	1	1	1
185	Piauí	non-cases	M	11.9	0	0		Lymphoma	0	0	0
186	MG	non-cases	M	62	0	0		Two or more options	0	0	0
187	Piauí	VL < 3 years	M	2.5	1	0		VL	1	1	1
188	BH	non-cases	M	9.8	0	0		Congenital spherocitis	0	0	0
189	Piauí	non-cases	M	20.9	0	0		Obstructive liver disease	0	1	0
190	MG	LV/HIV	M	30	1	1	5	VL	1	1	1
191	MG	non-cases	M	41	0	1		Secondary to HIV	0	0	0
192	Piauí	VL > 3 years and 1 month	M	12.9	1	0		VL	0	0	0
193	Piauí	VL < 3 years		2.3	1	0		VL	1	1	1
194	Piauí	VL > 3 years and 1 month	M	29.9	1	0		VL	1	1	1
195	MG	non-cases	M	26	0	1		Mycobacteriosis	0	0	0
196	Piauí	VL > 3 years and 1 month	M	34.10	1	0		VL	1	1	1
197	MG	LV/HIV	M	50	1	1	151	VL	1	1	1
198	MG	non-cases	M	25	0	0		Other Infectious Disease	1	1	1
199	MG	LV/HIV	M	33	1	1	217	VL	0	0	0
200	MG	non-cases	M	31	0	0		splenomegaly + arbovirus	0	0	0
201	Piauí	LV/HIV		45.5	1	1	469	VL	1	1	1
202	BH	VL < 3 years	M	0.1	1	0		VL	1	1	1
203	Piauí	VL < 3 years		1.4	1	0		VL	0	1	0
204	MG	VL > 3 years and 1 month	F	8.3	1	0		VL	1	1	1
205	BH	non-cases	M	7.1	0	0		liver abscess	0	0	0
206	Piauí	non-cases	M	5.9	0	0		Mixed malaria	0	0	0
207	MG	non-cases	F	35.11	0	0		Leukemia	0	0	0
208	MG	VL > 3 years and 1 month	F	55	1	0		VL	1	1	1
209	Piauí	VL < 3 years		0.5	1	0		VL	0	0	0
210	MG	non-cases	M	15.7	0	0		Meningitis	0	0	0
211	Piauí	LV/HIV		46.4	1	1	124	VL	1	1	1

212	BH	non-cases	M	12.4	0	0		Infective endocarditis	0	0	0
213	Piauí	VL > 3 years and 1 month	M	42.10	1	0		VL	1	1	1
214	MG	LV/HIV	M	65	1	1	138	VL	1	1	1
215	MG	LV/HIV	M	50	1	1	37	VL	0	0	0
216	MG	LV/HIV	M	36	1	1	38	VL	1	1	1
217	MG	non-cases	M	54.10	0	0		Bone marrow aplasia	0	0	0
218	MG	non-cases	F	41	0	0		cirrhosis	0	0	0
219	MG	non-cases	F	0.1	0	0		Sepsis	0	1	1
220	MG	VL > 3 years and 1 month	M	54	1	0		VL	1	1	1
221	MG	LV/HIV	M	59	1	1	203	VL	0	1	1
222	MG	LV/HIV	F	37	1	1	53	VL	0	0	0
223	Piauí	LV/HIV		59	1	1	261	VL	1	1	1
224	BH	non-cases	F	11.11	0	0		Idiopathic Thrombocytopenic Purpura	0	0	0
225	Piauí	VL < 3 years	M	2.3	1	0		VL	0	0	0
226	MG	LV/HIV	M	21	1	1	254	VL	0	0	0
227	Piauí	VL < 3 years	F	0.9	1	0		VL	0	1	1
228	Piauí	non-cases	F	43.1	0	0		Liver cirrhosis	1	0	0
229	Piauí	VL > 3 years and 1 month	M	22.12	1	0		VL	1	1	1
230	BH	VL < 3 years	F	1.7	1	0		VL	1	1	1
231	Piauí	non-cases	M	38.11	0	0		Urinary tract infection	0	0	0
232	Piauí	VL > 3 years and 1 month	M	31.8	1	0		VL	1	1	0
233	Piauí	VL > 3 years and 1 month	M	9.12	1	0		VL	1	1	1
234	MG	VL > 3 years and 1 month	F	3.12	1	0		VL	1	1	1
235	BH	VL < 3 years	F	2.9	1	0		VL	0	1	0
236	Piauí	VL < 3 years		2.4	1	0		VL	0	1	0
237	BH	VL < 3 years	F	0.7	1	0		VL	1	1	1
238	Piauí	VL < 3 years	M	0.9	1	0		VL	0	1	0
239	Piauí	VL > 3 years and 1 month	F	24.4	1	0		VL	1	1	1
240	MG	VL > 3 years and 1 month	M	44	1	0		VL	1	1	1
241	Piauí	LV/HIV		25.1	1	1		VL	0	0	0
242	Piauí	VL < 3 years		0.6	1	0		VL	1	1	1
243	MG	non-cases	M	40	0	1		Mycobacteriosis	0	0	0
244	MG	LV/HIV	M	39	1	1	39	VL	0	0	0
245	Piauí	VL > 3 years and 1 month	M	17.4	1	0		VL	1	1	1
246	Piauí	LV/HIV		46.1	1	1	159	VL	0	0	0
247	MG	non-cases	M	11.11	0	0		Lupus Erythematosus	0	0	0
248	MG	non-cases	F	31.12	0	0		Gastric adenocarcinoma	0	0	0
249	MG	LV/HIV	M	52	1	1	389	VL	0	1	1
250	Piauí	VL < 3 years		0.9	1	0		VL	0	1	1
251	MG	VL > 3 years and 1 month	M	44	1	0		VL	0	0	0
252	Piauí	VL < 3 years	M	2.8	1	0		VL	0	0	0
253	Piauí	VL > 3 years and 1 month	M	26.4	1	0		VL	1	1	1
254	Piauí	VL > 3 years and 1 month	M	6.3	1	0		VL	1	1	1
255	Piauí	non-cases	M	1.9	0	0		Encephalitis	0	0	0
256	MG	VL > 3 years and 1 month	M	52	1	0		VL	1	1	1
257	Piauí	VL < 3 years	M	1.1	1	0		VL	0	0	0
258	BH	non-cases	M	5.5	0	0		Leukemia	0	0	0
259	Piauí	VL > 3 years and 1 month	M	24.7	1	0		VL	1	1	1
260	MG	VL > 3 years and 1 month	M	68	1	0		VL	1	1	1
261	Piauí	non-cases	M	48.11	0	0		Adult Still's Disease	0	0	0
262	Piauí	LV/HIV		50.3	1	1	150	VL	0	0	0
263	Piauí	LV/HIV		38.9	1	1	393	VL	0	0	0
264	MG	VL > 3 years and 1 month	M	36	1	0		VL	1	1	1

265	Piauí	VL < 3 years	M	1.8	1	0		VL	1	1	1
266	Piauí	VL < 3 years	M	0.9	1	0		VL	1	1	1
267	Piauí	non-cases	F	3.4	0	0		Leukosis	0	0	0
268	MG	LV/HIV	M	30	1	1	267	VL	1	1	1
269	Piauí	non-cases	F	10.4	0	0		Malaria	0	0	0
270	MG	LV/HIV	M	25	1	1	39	VL	0	0	0
271	Piauí	VL < 3 years		2.7	1	0		VL	0	1	0
272	MG	LV/HIV	M	39	1	1	110	VL	0	0	0
273	MG	non-cases	M	1.1	0	0		Sepsis	0	0	0
274	Piauí	VL > 3 years and 1 month	M	48.1	1	0		VL	1	1	1
275	MG	VL > 3 years and 1 month	F	36.12	1	0		VL	1	1	1
276	MG	VL > 3 years and 1 month	M	32	1	0		VL	1	1	1
277	Piauí	VL > 3 years and 1 month	F	14.11	1	0		VL	1	1	1
278	Piauí	VL > 3 years and 1 month	F	19.3	1	0		VL	0	1	1
279	Piauí	VL < 3 years	F	1.1	1	0		VL	0	0	0
280	MG	non-cases	M	69.4	0	0		Lymphoma	0	0	0
281	BH	VL < 3 years	M	2.5	1	0		VL	1	1	1
282	Piauí	VL > 3 years and 1 month	M	49.1	1	0		VL	1	1	1
283	Piauí	VL < 3 years	F	2.6	1	0		VL	1	1	1
284	Piauí	LV/HIV		52.5	1	1		VL	1	1	1
285	BH	VL < 3 years	F	0.9	1	0		VL	0	0	0
286	MG	non-cases	M	48.11	0	0		Fungal Sepsis	0	0	0
287	Piauí	LV/HIV		67.2	1	1		VL	0	1	1
288	Piauí	VL > 3 years and 1 month	M	19.9	1	0		VL	1	1	1
289	Piauí	VL < 3 years		0.8	1	0		VL	1	1	1
290	Piauí	VL < 3 years	F	2.8	1	0		VL	1	1	1
291	BH	non-cases	F	7.3	0	0		Typhoid fever	0	0	0
292	BH	non-cases	M	0.12	0	0		Leukemia	0	0	0
293	Piauí	LV/HIV		37.1	1	1	152	VL	0	0	0
294	MG	non-cases	M	64	0	0		cytopenia + arbovirus	0	0	0
295	BH	VL > 3 years and 1 month	M	4.3	1	0		VL	1	1	1
296	MG	VL > 3 years and 1 month	M	42	1	0		VL	0	0	0
297	Piauí	VL > 3 years and 1 month	M	24.8	1	0		VL	1	1	1
298	MG	non-cases	M	10.1	0	0		Leukemia	0	0	0
299	MG	LV/HIV	M	51	1	1	50	VL	1	1	1
300	BH	VL < 3 years	M	2.8	1	0		VL	0	1	1

MG: Minas Gerais; BH: Bahia; M: Masculine; F: Feminine; VL: Visceral Leishmaniasis
