# Supplementary material for: Impact of age and immune status on the accuracy of rapid diagnostic tests for visceral leishmaniasis in Brazil
Source: PLoS Negl Trop Dis. 2025 Jun 2;19(6):e0013087. doi: 10.1371/journal.pntd.0013087 (PMC12129180; doi:10.1371/journal.pntd.0013087)
Supplement: S3 Appendix — (DOCX) [file pntd.0013087.s003.docx]

**S3 Appendix**. Agreement between the RDTs evaluated according to the subgroups

Overall agreement between the LSH Ab Eco Teste (Eco Diagnostica) and VH Bio (Quibasa) tests, by subgroup of interest.

| Overall agreement | | VH Bio | |
| --- | --- | --- | --- |
|  |  | Positive | Negative |
| LSH Ab Eco Teste | Positive | 154 | 14 |
|  | Negative | 2 | 130 |
| Kappa (CI95%) | | 0.893 (0.842 – 0.944) | |
| Agreement rate (%) (CI95%) | | 94.6 (91.5 – 96.7) | |
| Patients≤ 3 years | | VH Bio | |
|  |  | Positive | Negative |
| LSH Ab Eco Teste | Positive | 47 | 7 |
|  | Negative | 0 | 21 |
| Kappa (IC95%) | | 0.790 (0.645 – 0.935) | |
| Agreement rate (%) (CI95%) | | 90.7 (81.9 – 95.4) | |
| Patients> 3 years | | VH Bio | |
|  |  | Positive | Negative |
| LSH Ab Eco Teste | Positive | 69 | 1 |
|  | Negative | 1 | 4 |
| Kappa (CI95%) | | 0.786 (0.498 – 1.000) | |
| Agreement rate (%) (CI95%) | | 97.3 (90.8 – 99.3) | |
| HIV/VL coinfection | | VH Bio | |
|  |  | Positive | Negative |
| LSH Ab Eco Teste | Positive | 35 | 5 |
|  | Negative | 0 | 35 |
| Kappa (CI95%) | | 0.867 (0.756 – 0.979) | |
| Agreement rate (%) (CI95%) | | 93.3 (85.3 – 97.1) | |
| Non-cases | | VH Bio | |
|  |  | Positive | Negative |
| LSH Ab Eco Teste | Positive | 3 | 1 |
|  | Negative | 1 | 70 |
| Kappa (CI95%) | | 0.850 (0.562 – 1.000) | |
| Agreement rate (%) (CI95%) | | 97.3 (90.8 – 99.3) | |

Overall agreement between the LSH Ab Eco Teste (Eco Diagnostica) and Kalazar Detect (INBIOS International) tests, by subgroup of interest.

| Overall agreement | | Kalazar Detect | |
| --- | --- | --- | --- |
|  |  | Positive | Negative |
| LSH Ab Eco Teste | Positive | 166 | 2 |
|  | Negative | 14 | 118 |
| Kappa (CI95%) | | 0.891 (0.839 – 0.943) | |
| Agreement rate (%) (CI95%) | | 94.6 (91.5 – 96.7) | |
| Patients ≤ 3 years | | Kalazar Detect | |
|  |  | Positive | Negative |
| LSH Ab Eco Teste | Positive | 53 | 1 |
|  | Negative | 9 | 12 |
| Kappa (IC95%) | | 0.626 (0.422 – 0.830) | |
| Taxa de concordância (%) (IC95%) | | 86.7 (77.2 – 92.6) | |
| Patients > 3 years | | Kalazar Detect | |
|  |  | Positive | Negative |
| LSH Ab Eco Teste | Positive | 69 | 1 |
|  | Negative | 1 | 4 |
| Kappa (CI95%) | | 0.786 (0.498 – 1.000) | |
| Agreement rate (%) (CI95%) | | 97.3 (90.8 – 99.3) | |
| HIV/VL coinfection | | Kalazar Detect | |
|  |  | Positive | Negative |
| LSH Ab Eco Teste | Positive | 40 | 0 |
|  | Negative | 3 | 32 |
| Kappa (CI95%) | | 0.919 (0.830 – 1.000) | |
| Agreement rate (%) (CI95%) | | 96.0 (88.9 – 98.6) | |
| Non-cases | | Kalazar Detect | |
|  |  | Positive | Negative |
| LSH Ab Eco Teste | Positive | 4 | 0 |
|  | Negative | 1 | 70 |
| Kappa (CI95%) | | 0.882 (0.654 – 1.000) | |
| Agreement rate (%) (CI95%) | | 98.7 (92.8- 99.8) | |

Overall agreement between the VH Bio (Quibasa) and Kalazar Detect (INBIOS International) tests, by subgroup of interest.

| Overall agreement | | Kalazar Detect | |
| --- | --- | --- | --- |
|  |  | Positive | Negative |
| VH Bio | Positive | 153 | 3 |
|  | Negative | 27 | 117 |
| Kappa (CI95%) | | 0.798 (0.731 – 0.866) | |
| Agreement (%) (CI95%) | | 90.0 (86.1 – 92.9) | |
| Patients≤ 3 years | | Kalazar Detect | |
|  |  | Positive | Negative |
| VH Bio | Positive | 46 | 1 |
|  | Negative | 16 | 12 |
| Kappa (CI95%) | | 0.457 (0.259 – 0.654) | |
| Agreement rate (%) (CI95%) | | 82.9 (72.4 – 89.9) | |
| Patients> 3 years | | Kalazar Detect | |
|  |  | Positive | Negative |
| VH Bio | Positive | 69 | 1 |
|  | Negative | 1 | 4 |
| Kappa (IC95%) | | 0.786 (0.498 – 1.000) | |
| Agreement rate (%) (CI95%) | | 97.3 (90.8 – 99.3) | |
| HIV/VL coinfection | | Kalazar Detect | |
|  |  | Positive | Negative |
| VH Bio | Positive | 35 | 0 |
|  | Negative | 8 | 32 |
| Kappa (CI95%) | | 0.789 (0.653 – 0.924) | |
| Agreement rate (%) (CI95%) | | 89.3 (80.3 – 94.5) | |
| Non-cases | | Kalazar Detect | |
|  |  | Positive | Negative |
| VH Bio | Positive | 3 | 1 |
|  | Negative | 2 | 69 |
| Kappa (CI95%) | | 0.646 (0.274 – 1.000) | |
| Agreement rate (%) (CI95%) | | 96.0 (88.9 – 98.6) | |
